# Eosinophil Granule Proteins ECP and EPX as Markers for a Potential Early-Stage Inflammatory Lesion in Female Genital Schistosomiasis (FGS)

**DOI:** 10.1371/journal.pntd.0002974

**Published:** 2014-07-17

**Authors:** Charles Emile Ramarokoto, Anna Overgaard Kildemoes, Bodo Sahondra Randrianasolo, Pascaline Ravoniarimbinina, Vololomboahangy Elisabeth Ravaoalimalala, Peter Leutscher, Eyrun Floerecke Kjetland, Birgitte Jyding Vennervald

**Affiliations:** 1 Department of Epidemiology, Institut Pasteur de Madagascar, Antananarivo, Madagascar; 2 Section for Parasitology and Aquatic Diseases, Faculty for Health and Medical Sciences, University of Copenhagen, Copenhagen, Denmark; 3 Helminthiasis Unit, Institut Pasteur de Madagascar, Antananarivo, Madagascar; 4 Ministry of Public Health, Madagascar, Antananarivo, Madagascar; 5 Department of Infectious Diseases, Aarhus University Hospital, Aarhus, Denmark; 6 Norwegian Centre for Imported and Tropical Diseases, Department of Infectious Diseases, Oslo University Hospital Ulleval, Oslo, Norway; 7 School of Public Health Medicine, Nelson R Mandela School of Medicine, University of KwaZulu-Natal, Durban, South Africa; Stanford University School of Medicine, United States of America

## Abstract

**Background:**

Genital granulomas induced by *Schistosoma haematobium* eggs can manifest as different lesion types visible by colposcopy; rubbery papules (RP), homogenous sandy patches (HSP) and grainy sandy patches (GSP). Pronounced tissue eosinophilia is a candidate marker for active *S. haematobium* pathology, as viable schistosome egg granulomas often are eosinophil rich. Here it was investigated whether eosinophil granule proteins ECP (eosinophil cationic protein) and EPX (eosinophil protein-X) in urine and genital lavage can be used as markers for active FGS lesions.

**Methods:**

Uro-genital samples from 118 Malagasy women were analysed for ECP and EPX by standard sandwich avidin/biotin amplified ELISA.

**Principal findings:**

The women with RP lesions had significantly higher levels of ECP and EPX in both lavage and urine. Furthermore, women with RP lesions were significantly younger than those with GSP. This could indicate that RP lesions might be more recently established and thus represent an earlier inflammatory lesion stage.

**Conclusion:**

ECP in genital lavage might be a future tool aiding the identification of FGS pathology at a stage where reversibility remains a possibility following praziquantel treatment.

## Author Summary

The blood-dwelling fluke *Schistosoma haematobium* produce eggs which can inflict lesions both in the urinary and genital tract. Lesions in the female genital tract have been hypothesised to confer higher risk of contraction of HIV and other genital infections. These epithelial genital lesions are visibly different and three types can be observed; rubbery papules, homologous sandy patches and grainy sandy patches. Like other helminths, active *S. haematobium* infection is associated with eosinophilia. Therefore eosinophil granule proteins might be useful markers for active inflammation related to female genital schistosomiasis lesions. This study identifies the rubbery papules as a potential early-stage inflammatory lesion type associated with high levels of eosinophil granule proteins in vaginal lavage. It may be advantageous to identify female genital lesions relatively early after infection as chronic inflammation stage lesions might not respond to praziquantel treatment.

## Introduction

The poverty associated disease uro-genital schistosomiasis caused by *Schistosoma haematobium* affects millions of people in the Sub-Saharan region resulting in a substantial morbidity burden ranging from subtle to severe in individuals [1]. The main burden of morbidity ascribed to uro-genital schistosomiasis is not caused by the blood-dwelling mature flukes, but rather by the eggs they release inducing immune-related tissue reactions [2]. Presence of *Schistosoma haematobium* eggs not only in the urinary tract but also the genital tract tissues is far from a new finding, as it was first described in Egypt in 1899 [3]. Later several post-mortem studies have demonstrated that eggs occur just as frequently in genital tissues as in the urinary bladder, however possibly at a lower density [4]–[7]. Recently, a number of population-based epidemiological studies investigating genital manifestations of schistosomiasis in women, known as female genital schistosomiasis (FGS), have been carried out in various Sub-Saharan regions. These studies have demonstrated prevalence of FGS as high as 50% in *S. haematobium* endemic areas [8]. Research on various aspects of FGS has gained momentum as attention has been brought to hallmark features of the disease, namely disruption of the genital mucosal barrier, and local vascular and immunological changes. These factors might exacerbate the risk of contracting sexually transmitted diseases – of which HIV stands out as the most prominent consequence [9]–[11].

Approximately 112 million people are estimated to be infected with *S. haematobium* in the Sub-Saharan region of which a large proportion is at risk of having genital morbidity [12], [13]. FGS was added to the WHO gender task force in 1997 [14], [15]. Yet, assessments of the actual magnitude of FGS are easily prone to gross underestimations due to insufficient documentation and knowledge about the epidemiology and associated morbidity. This is underlined by the fact, that even if a woman is diagnosed by state-of-the-art colposcopy, FGS may be overlooked as eggs can be deposited in un-investigated sites further up in the genital tract or obscured by the anatomy in the fornices or on the vaginal walls [5], [6], [16]. Furthermore, women positive for FGS do not necessarily excrete eggs in urine enhancing the likelihood of underestimation of prevalence in areas where knowledge about FGS related morbidity is limited [17]. Colposcopy reveals visibly different FGS pathology: rubbery papules (RP), homogenous sandy patches (HSP), and grainy sandy patches (GSP) [10].

It has been well documented in studies focusing on the urinary tract that the *S. haematobium* egg induced granulomas are dominated by eosinophils [1], [18]–[20], hence tissue eosinophilia is a candidate marker for inflammatory activity in the genital lesions. The ability of eosinophils to kill helminths is ascribed to an array of basic cationic “attack” proteins packed in characteristic granules and amongst them are eosinophil protein-X (EPX, also known as eosinophil derived neurotoxin (EDN)) and eosinophil cationic protein (ECP) [21], [22]. The stability and constitutive presence of these proteins in eosinophils render them attractive candidates as measures of the degree of tissue eosinophilia, and thus potentially the inflammatory activity or severity associated with FGS lesions. It has previously been demonstrated that EPX and in particular ECP can be used as markers for bladder morbidity in urinary schistosomiasis [23]–[25]. Furthermore, earlier studies examining ECP in genital lavage have indicated a relation between elevated levels of this granule protein marker and FGS pathology, however the specific lesion type was not taken into account [26], [27]. This study aimed to investigate the potential of ECP and/or EPX as candidate markers for distinct FGS lesion pathology in the lower genital tract.

## Materials and Methods

### Study area and participants

A FGS case definition study was carried out in June–July 2010 in Miandrivazo district, Madagascar headed by the Institut Pasteur de Madagascar (IPM). The study participants were recruited from five villages in Miandrivazo district, which is located in the western part of Madagascar. Four of the chosen villages were identified as potential hyper-endemic villages (>50% *S. haematobium* prevalence) in previous surveys conducted in 2009 by IPM in collaboration with the schistosomiasis unit at the Madagascar Ministry of Health. These surveys showed a *S. haematobium* eggs in urine prevalence of 82% (61/74) in one village and 84% (89/106) based on pooled data from two high endemic villages. In the last high endemic village the prevalence of *S. haematobium* was based on observation of a high prevalence of macro-haematuria as reported by the local medical officer. The fifth selected village had low *S. haematobium* endemicity (<20% prevalence) based on an IPM survey from 1999 which found 8% (8/96) *S. haematobium* egg positive in urine. All five villages had comparable socio-economic levels and infrastructures. Inclusion criteria for study enrolment were 15–35 years of age, female gender, not having received anti-schistosomal treatment within the previous two years and having lived in the district for more than five years. Exclusion criteria were pregnancy, virginity, and eggs in urine status. One hundred and eighteen women, who wanted to participate and met the inclusion criteria, were recruited. Samples were obtained from 79 women from the four hyper-endemic villages (all egg positive). The control group consisted of samples from 39 women from the low endemic village matched by age to the women from the *S. haematobium* high endemic villages. Only control group individuals who did not shed eggs in the collected urine samples were recruited.

### Urine and genital lavage samples

Midstream urine samples were collected between 9am and 2pm on three consecutive days one of which coincided with the pelvic exam. A subsample of 10 ml urine was filtrated through a Nucleopore membrane immediately after collection and subsequently counted by standard light microscopy in the field laboratory. *S. haematobium* arithmetic mean egg counts (eggs/10 ml urine) were calculated based on the three urine samples. The urine sample for ECP and EPX level was collected on the day of the pelvic examination. An aliquot of 2 ml urine was immediately snap-frozen in liquid nitrogen. Urine samples were kept at −80°C and transported to the laboratory on dry ice. Furthermore, genital lavage was collected by spraying 10 ml 0.9% NaCl on the cervix and the inner half of the vaginal surfaces, whereafter it was pulled back into the syringe and re-sprayed. This was repeated four times in total. A set of 2 ml vials for ECP and EPX level determination were frozen and kept at −80°C from collection to the laboratory.

### Pelvic exam and STI assessment

As briefly outlined, FGS was diagnosed by colposcopy as previously described by Kjetland et.al. [10], [28] and lesions categorised as rubbery papules (RP), homogenous sandy patches (HSP), and grainy sandy patches (GSP). Women presenting with at least one of either lesion type were considered FGS positive.

Vaginal swabs were taken in order to test for a range of genital infections. The diagnostic methods used to supplement the clinical findings for diagnosis are given in brackets; *Trichomonas vaginalis* (microscopy of wet-mount and Pap-stained slides, PCR), *Neisseria gonorrhoea* (PCR), *Chlamydia trachomatis* (PCR), *Mycoplasma genitalium* (PCR), Human papilloma virus (High-risk-HPV PCR), *Candida albicans* (wet-mount microscopy, Pap-stained slides) and bacterial vaginosis (microscopy of wet-mount (Amsel's criteria) and Pap-stained slides). If a genital ulcer was found a swab was applied on the ulcer to test for Herpes simplex virus type 2 (PCR), and *Haemophilus ducreyi* (PCR). Furthermore cytobrush and spatula were used to collect material for Papanicolaou-stained slides in order to check for *S. haematobium* eggs and inflammatory signs. Lastly a serum sample was also tested for *Treponema pallidum* (RPR, TPHA).

### Enzyme-linked immunosorbent assay (ELISA)

Urine and genital lavage samples were analysed for ECP and EPX level in 2012 by standard avidin/biotin amplified sandwich ELISA using the protocol developed by Claus Reimert [29], [30] with minor alterations in the form of antibody and sample concentration adjustments. The antibodies used were the same as utilised by Reimert *et. al*. Standard curve detection range was 31–2000 pg/ml for EPX and 16–1000 pg/ml for ECP. Plates were read at 492 nm with a reference reading at 595 nm in ELISA-reader (Thermo Scientific Multiskan FC).

### Ethical considerations

Ethical permission was granted by the Committee of Ethics at the Ministry of Health in Madagascar (N° 031-CE/MINSAN 4 June 2010). A female physician informed the study participants in the local Malagasy language about the purpose and overview of the study, the procedures, the benefits and possible negative consequences of participation, privacy and confidentiality procedures, and the right to ask questions and to withdraw at any time point. The woman was asked for a signature if she accepted to participate in the study. Illiterate women who gave their free consent were asked to stamp their fingerprint. This was a procedure approved by the Committee of Ethics.

All women diagnosed with schistosomiasis were treated with a single dose of praziquantel (40 mg/kg body weight). If symptoms or signs of sexually transmitted infections or other diseases were observed, treatment or referral in accordance with the standard syndromic approach used in Madagascar was applied free of charge. Asymptomatic STIs were treated upon reception of STI testing results from the laboratory. Partner treatment was offered by a male doctor in a neighbouring location. All study participant information was anonymised and securely stored.

### Data analysis

**Figure 1:**
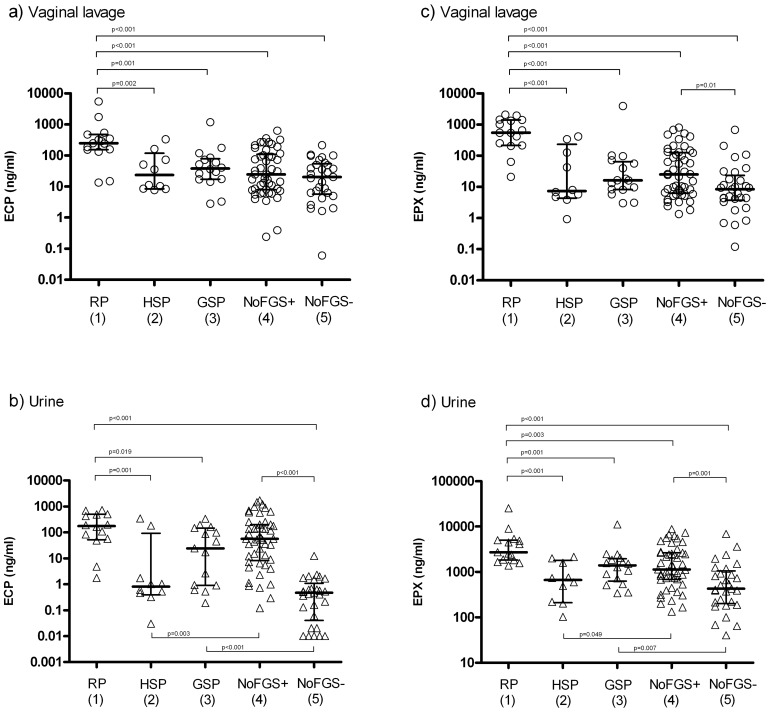
Dot plot illustrating the amount (ng/ml) of ECP in genital lavage (a) and in urine (b) and of EPX in genital lavage (c)) and urine (d) classified by pathology category 1–5 (see table 1). The plus and minus following “NoFGS” in group 4 and 5 refer to the *S. haematobium* egg status. The median is indicated with a horizontal bar and interquartile ranges shown. Only significant p values are shown on the graphs. For ECP in urine two extreme outlies were excluded (b). Note that the ordinate axis is on logarithmic scale.

ELISA absorbance readings and protein concentration calculations were handled using SkanIt3.1.0.4 software. Only standard curves with a correlation co-efficient (r) of minimum 0.95 were accepted, and protein concentrations were determined using linear regression. Data were analysed using SPSS 19.0.0 for Windows (IBM SPSS Inc.), and the graphical representation in figure 1 was made using GraphPad Prism 4.03 software. Spearman's correlation co-efficient was used to describe the relation between granule proteins and mean egg counts. Two-tailed non-parametric Mann-Whitney U (2-sample) and Kruskall-Wallis (k-sample) tests were used to test for significant differences in protein levels in independent samples. In order to test independent variables for significance, multinominal logistic regression models using pathology categories as dependent variable were run (protein levels were normally distributed after log transformation). Table 1 shows the lesion category classification used for data analysis.

**Table 1:** FGS diagnostic categories used for data analysis.

Category:	1 (RP)	2 (HSP)	3 (GSP)	4 (NoFGS eggpos)	5 (NoFGS eggneg)
Rubbery papule (RP)	**√**	**-**	**-**	**-**	**-**
Homogenous sandy patch (HSP)	**?**	**√**	**-**	**-**	**-**
Grainy sandy patch (GSP)	**?**	**?**	**√**	**-**	**-**
Urine egg positive (n)	**15**	**2**	**14**	**48**	**0**
Urine egg negative (n)	**0**	**8**	**2**	**0**	**29**
Total n	**15**	**10**	**16**	**48**	**29**
Mean (median) age	**19 (19)**	**23 (22)**	**25 (24)**	**22 (20)**	**23 (21)**
Age range (yrs)	**15–25**	**15–35**	**16–32**	**15–33**	**15–35**
Mean (median) egg count	**262 (125)**	**15 (0)**	**134 (9)**	**346 (35)**	**0 (0)**
Egg count range	**2–1091**	**0–74**	**0–877**	**1–2463**	**0**

**√**: At least one manifestation of the pathology type in question.

**?**: The pathology can be present.

**-** : No manifestations of the given type.

Below the category number in brackets are the terms used in the text and on figure 1 for each grouping.

### Accession numbers

UniProtKB/Swiss-Prot

ECP: P12724

EPX: P10153

## Results

Women from the hyper-endemic villages (n = 79) had *S. haematobium* egg counts in the range from 1–2463 eggs/10 ml urine with an geometric mean intensity of 35 eggs/10 ml urine and a median of 51 eggs/10 ml urine. Gynaecological examinations showed that 41 of the total 118 women were FGS positive (34.7%), whereas 77 had neither a rubbery papule (RP), homogenous sandy patch (HSP), nor a grainy sandy patch (GSP) on the vaginal or cervical mucosal surface visible by colposcopy as described in Kjetland *et.al.* 2005. Women from the low endemic village (n = 39) were recruited based on being *S. haematobium* egg negative in urine, however, 10 women (25.6%) from this group were found FGS positive (table 1). No urine egg negative women had RPs. The FGS prevalence in women from the high endemic villages was 39.2%. Mean urine egg count was significantly lower (p = 0.04) in FGS positive compared to FGS negative (FGS negative egg negative excluded, cat.5). No significant difference in mean urine egg count was found when comparing the RP group with the GSP group (p = 0.054). However, HSP positive women had significantly less eggs in urine compared to RP positive (p<0.001) and GSP positive (p = 0.007).

The age span of the 118 women was from 15 to 35 years of age and there was no significant difference in age between FGS positives and negatives (p = 0.719). However, women with only GSP lesions were significantly older than RP positive women (p = 0.007).

### Eosinophil cationic protein (ECP)

FGS positive women had a median level of 72.5 ng/ml (range 2.79–5480 ng/ml) of ECP in genital lavage. This significantly (p = 0.002) exceeded the ECP level found in genital lavage in the FGS negative women of 24.4 ng/ml (range 0.06–625 ng/ml). In urine FGS positive women had a median ECP level of 56.1 ng/ml (range 0.03–1.27^12^ ng/ml), which was also higher than the level in FGS negative women of 6.4 ng/ml ECP (range 0.01–1720 ng/ml). However, this difference was not significant (p = 0.056). Positive correlations were found between the mean *S. haematobium* egg count in urine and the levels of ECP in both genital lavage and urine (p<0.01). However, the correlation was weaker in genital lavage (r = 0.257) as compared to urine (r = 0.773).

### Eosinophil protein X (EPX)

The median level of EPX in genital lavage was 63.5 ng/ml (range 0.92–3950 ng/ml) in FGS positive women, which was significantly (p = 0.003) higher, than the median level found in the FGS negative group of 10.6 ng/ml for EPX (range 0.12–810 ng/ml). In urine from FGS positive women the median level of EPX was 1650 ng/ml (range 103–25400 ng/ml). FGS negative women had a median level of 875 ng/ml (range 40–8840 ng/ml) in the urine, which was significantly lower compared to the FGS positive women (p = 0.024). EPX levels in both genital lavage and urine were positively correlated to the mean *S. haematobium* egg count in urine (p<0.01), but the strongest correlation was found in urine (r = 0.550) compared to genital lavage (r = 0.403).

### Elevated ECP and EPX in women with rubbery papule lesions

In order to investigate whether the significantly elevated ECP and EPX levels found in women with FGS were associated with a particular type of lesion pathology, analysis was done stratified by lesion categories (table 1). A significant difference between pathology categories was found for both median ECP (lavage p<0.001 and urine p = 0.001) and EPX (lavage p<0.001 and urine p<0.001) levels. Significantly higher levels of ECP found was found in genital lavage and urine in RP positive women compared to HSP positives, GSP positives and FGS negative egg negative women as shown in Figure 1a and 1b. However, the ECP level in RPs was only significantly higher in genital lavage and not in urine when comparing to the FGS negative egg positive group (p00.08). Almost the same pattern applied to EPX where the protein concentration also was significantly higher in genital lavage and urine in individuals with RP pathology compared to HSP positives, GSP positives and both categories of FGS negative women (Figure 1c and 1d).

Multinominal logistic regression show that having RP pathology encompasses a three-fold risk of elevated ECP (OR = 3.1; 95% CI[1.7–5.5]) and EPX (OR = 3.4; 95% CI[1.8–6.1]) levels in genital lavage. The regression analysis was controlled for presence of other genital infections (*Trichomonas vaginalis*, *Mycoplasma genitalium*, *Chlamydia trachomatis*, *Neisseria gonorrhoea*, bacterial vaginosis, *Candida albicans* or high-risk Human papilloma virus) and age. Test for *Haemophilus ducreyi* and Herpes simplex virus type 2 were only carried out if a genital ulcer was suspected (n = 3, no positives), hence these variables could not be taken into account.

## Discussion

The central question here was whether the eosinophil granule proteins ECP and/or EPX are suitable as identifiers for inflammatory FGS lesions. The results from this study showed an elevated level of EPX and ECP in genital lavage in association with the rubbery papule (RP) type lesion. In contrast sandy patches (grainy or homogeneous) were not associated with significantly higher granule protein levels in genital lavage. This could indicate that RPs represent eosinophil rich lesions, whereas GSPs and maybe HSPs might represent a later stage of the disease or at least a different lesion type in terms of inflammation. This corresponds with previous findings showing that high ECP levels in lavage were associated with FGS [27] and that ECP levels decreased in response to praziquantel treatment [26] although none of these studies took lesion type into consideration.

The likelihood of contracting schistosomiasis depends on water contact. Infection intensity measured by urinary egg output normally peaks in 10–14 year olds where after it declines in adults depending on the level of transmission [31]. The observation, that women with GSPs were significantly older than those with RPs, could hence reflect a difference in lesion type based on younger women harbouring active infections with higher infection intensities rather than different age-dependent pathology patterns. Furthermore, the tendency of higher urine egg count in RP positive women compared to GSP positive strengthen this viewpoint. These observations support that the GSP lesion represent a later stage lesion with calcified eggs and less marked inflammation. Comparable lesions have been described in the urinary bladder [1], [32], [33]. A study of Zimbabwean women from 20 to 49 years of age, who did not portray RPs at all, showed no resolution of GSP and HSP despite repeated praziquantel treatment [34] indicating a chronic type lesion pathology. If different lesions in fact reflect a difference in time of egg deposition rather than lesions with different inflammatory profiles per se, it is very likely that reversibility after treatment is only possible in early-stage inflammatory lesions. Regression of pathology after praziquantel treatment has been shown in several ultrasound based studies of the urinary tract as well as an interim report indicating a varying treatment effect on sandy patches in the genital tract [35]–[39]. However, it remains to be investigated whether RPs respond to treatment. It would be very relevant to determine whether ECP/EPX levels decrease after praziquantel treatment depending on pathology type.

If RPs are in fact early-stage lesions, it is very likely that they contain viable *S. haematobium* eggs, which excrete antigen. Presence of antigen may spark local immune reactions resulting in activated endothelial cells and chemotactic recruitment of a range of immune cells. *In vitro* studies have demonstrated that soluble egg antigens (SEA) from *S. mansoni* eggs induce endothelial cell proliferation and activation and it is possible that viable *S. haematobium* eggs in a similar manner induce vascular activity [40], [41]. Characteristic for *S. haematobium* granulomas containing viable eggs is the recruitment of high eosinophil numbers, but also enhanced macrophage, fibroblast and endothelial cell activity has been demonstrated [42]–[44]. As RPs possibly contain viable eggs which release antigen, they might show similar tissue activity as lesions described previously. This is indicated in a study of cervico-vaginal biopsies from Malawian women which found a positive association between viable parasite ova and granulation tissue rich in sprouting blood vessels as compared to normal healthy tissue and to tissue containing calcified ova [44]. A recent case report of a genital inflammatory lesion which may resemble rubbery papule was found to contain viable *S. haematobium* eggs [45].

Some caution is necessary when interpreting ECP/EPX levels as measures for tissue eosinophilia related to *S. haematobium* egg induced RP pathology, since basophils and particularly neutrophils occasionally might present a bias as source of the granule proteins [46]–[49]. This is especially relevant if strong bacterial co-infection or allergic responses are present [50], [51]. Furthermore, research is needed in order to establish whether some genital tract mucosal immune responses dominated by neutrophils might show significant levels of ECP and EPX, although it is generally established that eosinophils and not neutrophils are associated with helminth infections [52]–[54].

Both ECP and EPX protein levels were higher in association with RPs in genital lavage, but ECP in appears to be the better candidate marker, since EPX levels were higher in urine than in genital lavage even in the control group. Likewise, previous reports on urine samples from Kenyan children point towards ECP being superior to EPX as eosinophil marker protein for *S. haematobium* infection [24]. Here this observation is expanded to include genital lavage samples.

Studies conducted in Egypt, Ghana, Madagascar, Malawi, South Africa and Zimbabwe point towards a significant and under-recognised FGS related morbidity which may manifest as diversely as pelvic pain, infertility, a 3-fold increased risk of HIV as well as various social consequences [55]–[59]. FGS often present with symptoms, which are generally difficult to quantify and obtain accurate information about, such as vaginal discharge, mucosal contact bleeding, dyspareunia, pelvic pain and genital itch [28], [56], [60], [61]. Further complicating the diagnostic picture is an overlap of symptoms which are common in STDs [10], [62]. There is thus a need for non-invasive diagnostic research tools for investigating the epidemiology of FGS and efficacy of treatment particularly in young women. This study identifies ECP in genital lavage as a marker for a potential early-stage inflammatory FGS pathology. Combining this measure with testing for presence of eggs or current infection by schistosome PCR [63] or circulating anodic antigen (CAA) [64], [65] would further improve the diagnostic potential. This research tool-set could be used to expand our knowledge of the FGS prevalence and pathogenesis but most importantly aid in identifying pathology at a stage, where reversibility remains a possibility. Furthermore, it can shed light on the role of eosinophils in tissue inflammation in uro-genital schistosomiasis.
